# In Situ Dry Chemical Synthesis of Nitrogen-Doped Activated Carbon from Bamboo Charcoal for Carbon Dioxide Adsorption

**DOI:** 10.3390/ma15030763

**Published:** 2022-01-20

**Authors:** Weijun Ying, Shuo Tian, Huan Liu, Zenan Zhou, Grantson Kapeso, Jinhuan Zhong, Wenbiao Zhang

**Affiliations:** 1 National Engineering and Technology Research Center of Wood-Based Resources Comprehensive Utilization, Zhejiang Agriculture and Forestry University, Hangzhou 311300, China; wjying@zafu.edu.cn (W.Y.); ts0048@stu.zafu.edu.cn (S.T.); inspirelh@stu.zafu.edu.cn (H.L.); zhouzenan@stu.zafu.edu.cn (Z.Z.); grantksps@gmail.com (G.K.); 2 Jiyang College, Zhejiang Agriculture and Forestry University, Shaoxing 311800, China

**Keywords:** bamboo charcoal, bamboo-based activated carbon, N-doping activation, CO_2_ adsorption, capture capacity

## Abstract

In this work, nitrogen-doped bamboo-based activated carbon (NBAC) was in situ synthesized from simply blending bamboo charcoal (BC) with sodamide (SA, NaNH_2_) powders and heating with a protection of nitrogen flow at a medium temperature. The elemental analysis and X-ray photoelectron spectra of as-synthesized NBAC showed quite a high nitrogen level of the simultaneously activated and doped samples; an abundant pore structure had also been determined from the NBACs which has a narrow size distribution of micropores (<2 nm) and favorable specific surface area that presented superb adsorption performance. The fcarbon dioxide (CO_2_) adsorption of the NBACs was measured at 0 °C and 25 °C at a pressure of 1 bar, whose capture capacities reached 3.68–4.95 mmol/g and 2.49–3.52 mmol/g, respectively, and the maximum adsorption could be observed for NBACs fabricated with an SA/BC ratio of 3:1 and activated at 500 °C. Further, adsorption selectivity of CO_2_ over N_2_ was deduced with the ideal adsorbed solution theory ((IAST), the selectivity was finally calculated which ranged from 15 to 17 for the NBACs fabricated at 500 °C). The initial isosteric heat of adsorption (Qst) of NBACs was also determined at 30–40 kJ/mol, which suggested that CO_2_ adsorption was a physical process. The results of ten-cycle adsorption-desorption experimentally confirmed the regenerated NBACs of a steady CO_2_ adsorption performance, that is, the as-synthesized versatile NBAC with superb reproducibility makes it a perspective candidate in CO_2_ capture and separation application.

## 1. Introduction

Carbon dioxide (CO_2_) emission is extensively known as the reason for climate change and global warming [1]; international protocols and countermeasures have declared to achieve carbon neutrality [2]. Vast work among the academic community has been taken to alleviate the negative effect of the rapid growth of atmospheric CO_2_ concentration at a global level, such as developing renewable and clean energies [3,4,5] and functional porous materials [6,7], and decades of research and projects on carbon capture, utilization and sequestration (CCUS) [8,9,10] have been conducted to reduce the influence of carbon emission. And various solid absorbents, such like activated carbon (AC) [11,12,13,14], molecular sieve [15,16], metal oxides [17] and MOF [18], Among these products, ACs have been widely applied in carbon dioxide capture due to their special pore structure, specific surface area and chemical stability, and simple processing; tremendous research efforts have focused on the adsorption capacity, selectivity and renew-ability of activated carbon products [19,20,21,22,23,24].

Activated carbon can be synthesized from multiple bioresources by chemical activation. Idrees et al. [25] reported that peanut shell-deprived AC by KOH activation featured micropores (<1 nm) and the results showed that the structure had a positive relationship with CO_2_ adsorption. Modifications can further improve the adsorbing performance. For example, various reports [7,26,27,28] have demonstrated that sodamide activation was a useful approach to synthesize functionalized porous carbon materials for CO_2_ capture, and the nitrogen-doping method has been reported to be a versatile route for enhancing CO_2_ adsorption. For example, nitrogen functionalized biochar has been applied as a renewable adsorbent for efficient CO_2_ removal whose adsorption could reach up to 4.58 mmol/g and it was also found that the adsorption mainly rested with a micropore smaller than 0.80 nm [29]. N-doped AC prepared by urea and KOH co-activation using sugarcane waste [30] demonstrated a doubled CO_2_ capture capacity (4.8 mmol/g) as compared with an untreated control trial sample. Other materials, such as chitosan [31], glucose [32], and their derivatives are also employed as nitrogen sources for doping AC to obtain improved CO_2_ adsorption. It is essential to develop a cost-effective, commercially available raw material and an activation approach to prepare AC with high efficiency performance for a specific purpose.

Fast-growing bamboo is extensively cultivated across tropical and temperate regions which makes its value-added production sustainable around the world. Bamboo charcoal (BC) is the solid product of the pyrolysis of bamboo materials in the absence of oxygen. It is commercially available at any time in the market. The gaseous and liquid adsorption performance of bamboo charcoal and bamboo activated carbon (BAC) have been widely studied because the emerging BC/BAC has shown great potential in environmental purification. Specially designed, synthesized, and modified BC or BAC are applied in air quality improvement to remove formaldehyde [33], volatile organic compounds [34], carbon dioxide [35], sulfur dioxide, and nitrogen oxides [36], or in eradication of contaminants such as heavy metals in water [37] and antibiotics [38,39] in the pharmaceutical industry, and the wastes and leftovers of N-/P-modified bamboo charcoals are valid for soil amelioration [40] and carbon sequestration. Modified bamboo-based activated carbons prepared from bamboo and its processing residue are also used as CO_2_ absorbents [41,42] for their favorable adsorption performance.

However, either phosphoric acid or alkali activation to prepare activated carbon can be harmful to the environment or cause corrosion to equipment [43]; therefore, identifying activation materials of low pollution and corrosion is important to improve conventional processes. Traditional modifications generally require tedious processing and skilled work with high costs; therefore, developing a convenient synthesis of doped AC is beneficial to both industrial and academic research. In this work, a new method is presented to prepare N-doped BAC (NBAC) by a facile one-step in situ dry chemical process by simply blending bamboo charcoal with sodamide (NaNH_2_) in a tubular furnace activated at medium temperature (400–600 °C), which is much lower than that of conventional chemical activation that generally goes to 800 °C or beyond. The study results also showed that N-doped BACs had potential applications in CO_2_ capture and separation.

## 2. Results and Discussion

### 2.1. BET Characterization

**Figure 1:**
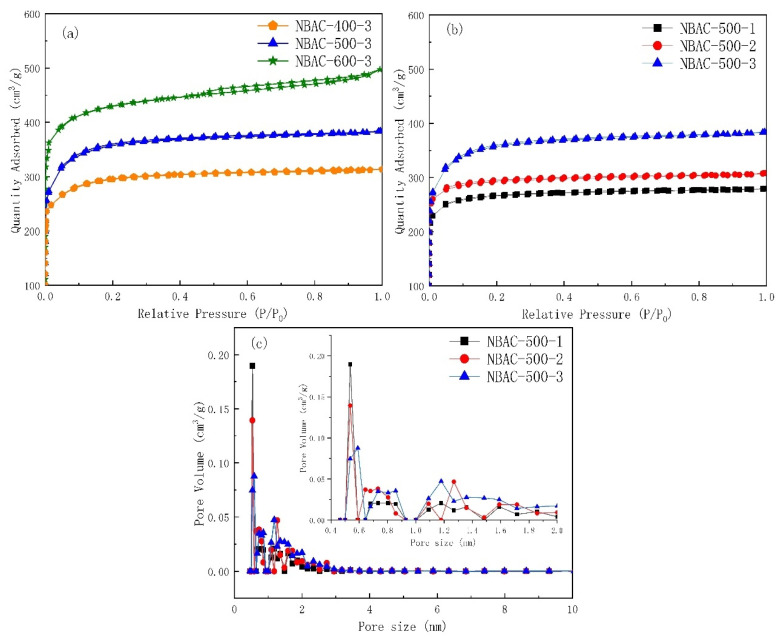
N_2_ adsorption–desorption isotherm (**a**,**b**) of N-doped bamboo-based activated carbon synthesized by sodamide activation and its pore size distribution (**c**).

N_2_ adsorption–desorption isotherm of NBAC was determined (Figure 1), which showed the adsorption performance of NBAC synthesized under controlled activation temperatures and NaNH_2_ (SA)/BC ratios. The achieved I type isotherm indicated that the as-synthesized samples had an abundant micropore (<1 nm) structure. When SA/BC were blended at a ratio of 3:1, it can be seen that, as the activation temperature increased from 400 °C to 600 °C, the isotherm results gradually increased, proving the corresponding increment of total pore volume and N_2_ adsorption capacity. When activated at 500 °C, the isotherm of NBAC almost reached a plateau at a relative pressure of 0.05, although no apparent hysteresis phenomenon appeared in that the dominated micropores were distributed in the range from 0.4–0.9 to 1.0–3.0 nm, as shown in Figure 1c; the results also implied that other NBACs obtained from different blend ratios had pores with a narrow pore size distribution. Notably, when activated at 600 °C, both the pore volume and size distribution were relatively small, nonetheless it is slightly broader than its counterparts obtained at 400 °C or 500 °C, respectively, which may have accounted for the visually distinguishable hysteresis phenomenon that occurred to NBAC-600s, as shown in Figure 1a.

When activated at 500 °C, the BACs obtained from a low SA dosage presented that the isotherm gradually augmented with an increase in the SA/BC ratio, as seen in Figure 1b. The isotherm reached saturated adsorption at a fairly low relative pressure. When an increased dosage of SA was used in activation, a sharp enhancement in the isotherm performance occurred, most notably in the range of low relative pressure, however, it disclosed a leveling off beyond a relative pressure (P/P_0_) of 0.2, which was also confirmed by the wide pore size distribution of micropores, as seen Figure 1c. Therefore, we concluded that a high SA dosage in activation may not be conducive to micropore-structured NBAC synthesis because the fierce activation could jeopardize micropore forming, causing neighboring micropores to breakdown or collapse into larger pores, and as a result, the synthesized NBAC would be less active in adsorbing small molecules such as CO_2_.

The results of specific surface area (S_BET_), total pore volume (V_tot_), micropore volume (V_mic_), and narrowly-distributed (0.33–1.0 nm) micropore volume (V_0.33–1_) are listed in Table 1. NBACs obtained from a low dosage of SA at 500 °C and below could be beneficial to CO_2_ adsorption, although the micropore volume of NBACs obtained from a high dosage of SA tended to decline at 600 °C, owing to the excessive temperature and activation overdose that accelerated pore reaming. In fact, it jeopardized new pore structure formation, thus a negative growth for micropores that ultimately unveiled in measurement results.

### 2.2. Morphological Analysis

**Figure 2:**
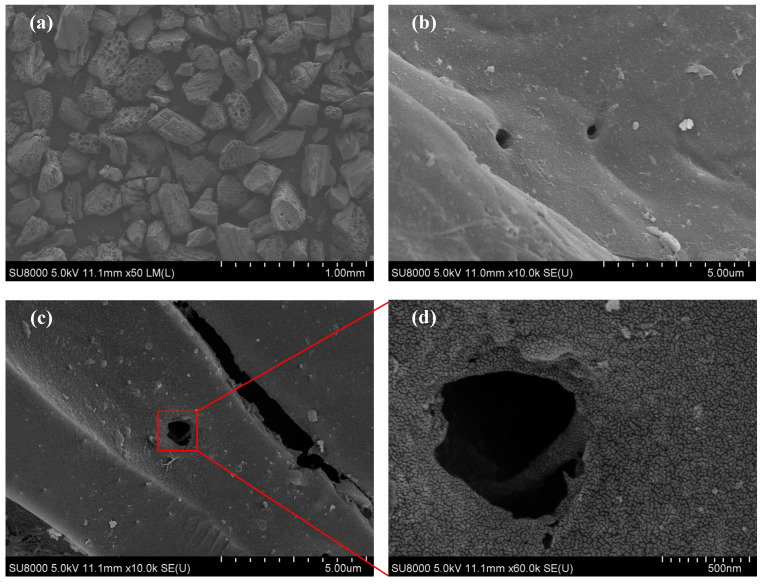
SEM patterns of bamboo charcoal (**a**,**b**) and N-doped bamboo-based activated carbon (**c**,**d**).

The microstructure patterns of NBACs were observed using a scanning electron microscope and are shown in Figure 2. Original porous bamboo morphologies were observed from the charcoal, under same low magnification (Figure 2b,c); there was no obvious surface difference between BC and BAC. Further enlargement completely exposed that the smooth surface of BAC was suffused with massive trenches and holes. The chemical etching by sodamide was effective and efficient in porosity generation. Especially, the occurrence of deep activation was observed through the hole structure that originated from the pits distributed on the vessel of bamboo, and provided a fair approach to augment surface area, therefore, making adsorption technically feasible.

### 2.3. Elemental Analysis

An elemental analysis was employed to explore the composition change before and after the dry chemical processing. As shown in Table 2, the nitrogen (N) content of untreated BC is approximately 0.26%, meanwhile, the activated/doped samples average N content is 10 times more than that of the untreated BC. Simply put, an increase in the nitrogen content of BAC signaled the successful modification of samples.

### 2.4. XPS Analysis

**Figure 3:**
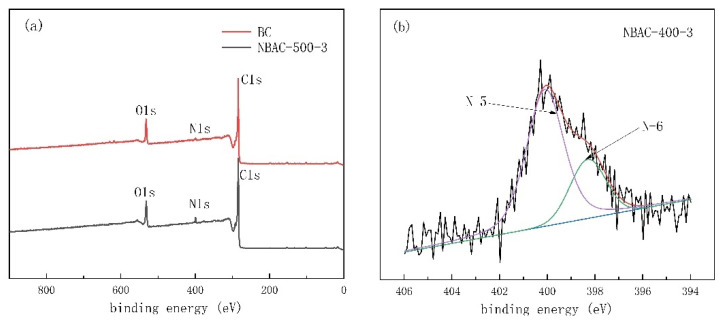

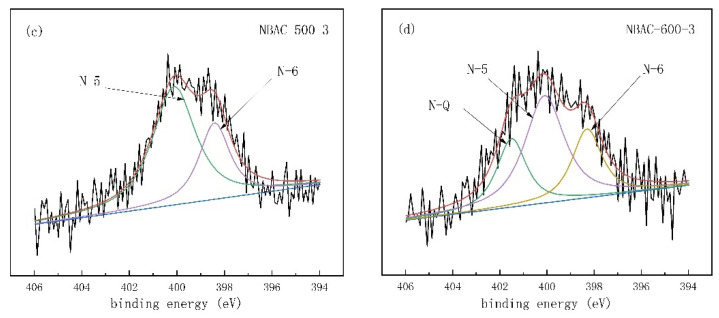
XPS spectra of NBAC-500-3 (**a**) and fitting curves of NBAC-400-3 (**b**), NBAC-500-3 (**c**) and NBAC-600-3 (**d**) synthesized from dry chemical activation.

The XPS spectra of BC and NBAC, as shown in Figure 3a, exhibited characteristic peaks (binding energy) at 285, 399, and 532 eV, attributed to C1s, N1s, and O1s, respectively; nevertheless, a comparative strong intensity of N1s peak of NBAC-500-3 stood out.

Accordingly, peak-differentiating and imitating of the raw XPS spectra of nitrogen atoms was successfully analyzed. The peaks at 398.3 and 400.1 eV can be assigned to the binding energy of pyrrolic N (N-5) and pyridinic N (N-6), respectively, as shown in Figure 3b–d. As shown in Figure 3d, an additional quaternary N (N-Q) at 401.5 eV emerged when samples were synthesized at 600 °C, supporting that the partial N-5 and N-6 phases were transforming toward the more thermodynamically stable N-Q phase, which coincided with that reported by [30]. Notably, N-5 had a favorable interaction with CO_2_ molecules [44] and, based on the XPS data, doping activation at 500 °C or below can better induce functional groups that facilitate CO_2_ capture. Thus, NBAC synthesized at 500 °C was, hereinafter, chosen as the object of study to explore the CO_2_ capture performance.

### 2.5. CO_2_ Adsorption Analysis

**Figure 4:**
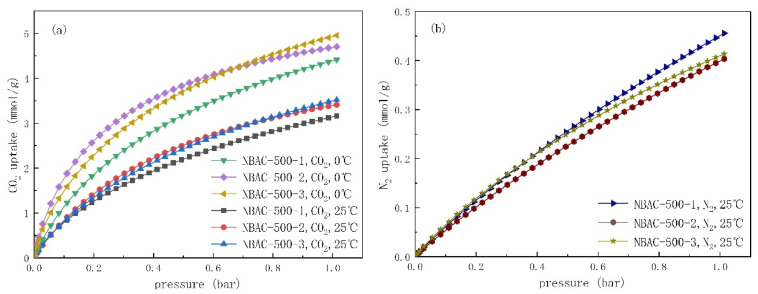
Adsorption isotherms of CO_2_ (**a**) and N_2_ (**b**) for NBACs synthesized at 500 °C.

The isotherm adsorption of CO_2_ and N_2_ of NBACs are shown in Figure 4a,b, respectively. It can be seen that, one the one hand, the adsorption capacity was prone to decline as the temperature increased, which was a remarkable feature of a physical adsorption. On the other hand, the capacity rose constantly, even when the pressure went beyond 1 bar, which demonstrated that the BAC would continue to adsorb CO_2_ or N_2_ at a higher pressure. In addition, the CO_2_ adsorption capacity was far higher than that of N_2_, based on the collected data. For that matter, the NBAC samples also outperformed the CO_2_ adsorption of three typical commercial BACs whose capacity varied from 1.43 mmol/g to 2.21 mmol/g, according to the authors’ laboratory measurements at an ambient temperature (25 °C), as indicated in Table 3.

The CO_2_ and N_2_ uptakes of BAC with pressure at 1 bar and temperature at 0 °C and 25 °C, respectively, are shown in Table 3. The uptake ranged from 3.68 to 4.95 mmol/g at 0 °C, and from 2.49 to 3.52 mmol/g at 25 °C, among which the maximum uptake occurred for the sample obtained with high SA activent dosage (BAC-500-3). The N_2_ uptake of all samples ranged from 0.33 to 0.49 mmol/g at 25 °C, which was much lower than that of CO_2_ uptake with the same conditions. It also found that with more dosage of activent at 500 °C or below the CO_2_ uptake of corresponding BAC were improved. It decreased as activent dosage went higher when activated at 600 °C.

It has been reported that CO_2_ uptake of N-doped porous carbon can be simultaneously influenced by a narrow pore size distribution of micropores and N content [45]. In this work, it was also found that it had an above-average level upon further investigation of the data in Table 1, Table 2 and Table 3. The maximum CO_2_ uptake reached 4.95 mmol/g and 3.52 mmol/g at 1 bar, at 0 °C and 25 °C, respectively, and NBAC-500-2 presented maximum narrow size distributed pores.

### 2.6. Analysis for Selectivity of CO_2_ over N_2_

The NBAC-500 samples were selected to explore their adsorption selectivity for CO_2_ capture in order to assess the dynamic adsorption behavior of mixture gas containing 15 vol.% CO_2_ and 85 vol.% N_2_, which is a representative proportion of flue gas. The isotherm was obtained by the Langmuir–Freundlich equation (Equation (1)) from the isotherm value of CO_2_ and N_2_ at 1 bar and 25 °C, and adsorption selectivity could be finally deduced in accordance with the ideal adsorbed solution theory (IAST, Equation (3)). The coefficient R^2^ values achieved 0.99 which showed good fitting; all detailed data and selectivity are summarized in Table 4. The selectivity of CO_2_ over N_2_ for NBACs was calculated to be between 15 and 17 at 25 °C, respectively. It seemed that the N content of NBAC (see Table 2) had a slightly positive effect on adsorption selectivity, which might be optimized in future works.

**Figure 5:**
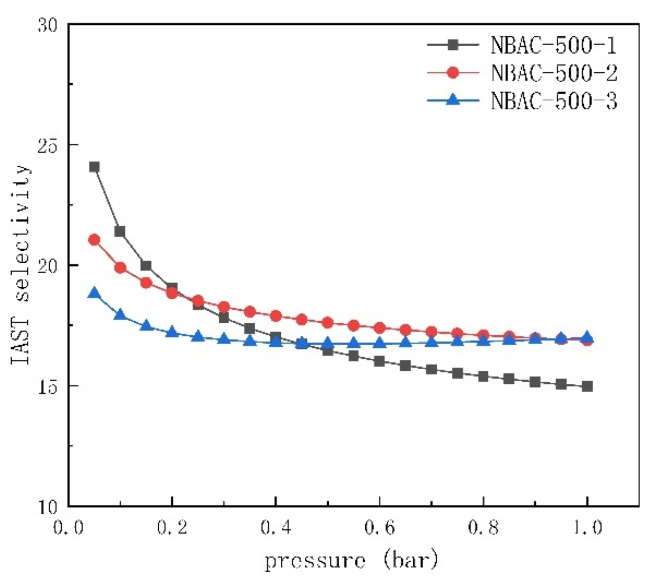
Adsorption selectivity of CO_2_ over N_2_ for NBAC synthesized at 500 °C.

The selectivity of NBAC is shown in Figure 5. It showed that optimal performance could be reached at a low pressure in that there were adequate adsorptive spots for CO_2_ capture, whereas higher pressure made the N-doped BAC relatively less selective to separate CO_2_.

### 2.7. Analysis for Isosteric Heat of Adsorption

**Figure 6:**
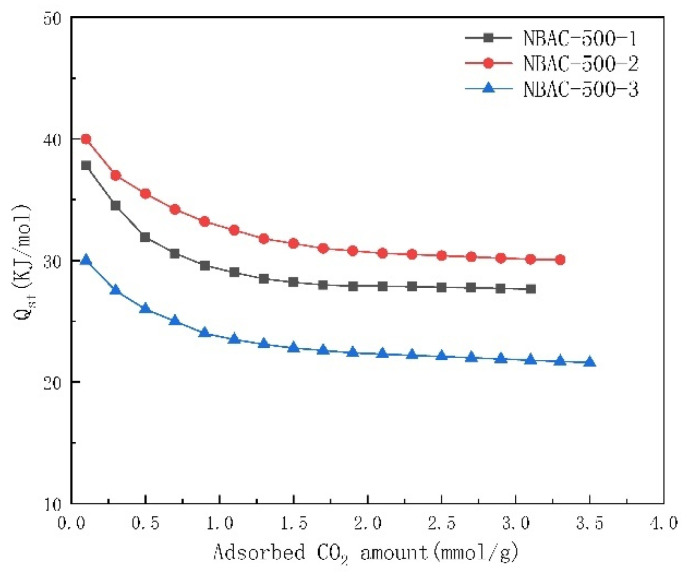
Isosteric heat of adsorption for NBACs synthesized at 500 °C.

An analysis of isosteric heat of adsorption is important to evaluate the adsorption performance of an absorbent; it provides the interaction information between absorbent and adsorptive. In this paper, the isosteric heat of adsorption (Qst) at 0 °C and 25 °C was determined in accordance with the Clausius–Clapeyron equation (Equation (2)). The values are illustrated in Figure 6. Should The initial CO_2_ adsorption approach “0” when epitaxy method applied to the current isotherm, the initial Qst shall be 30–40 KJ/mol, a typical value of physical adsorption that proved superior adsorptive performance of NBACs in this work. A low Qst causes the NBACs to have less energy consumption during the process of desorption, that is, it is more kinetically feasible to regenerate NBACs, which helps to reduce recycling costs. As the CO_2_ capture continued, the Qst had a tendency to decrease and stabilize, which may have been due to the topological non-uniformity and adsorption saturation of the NBAC samples.

### 2.8. Analysis for Reproducibility of CO_2_ Adsorption

**Figure 7:**
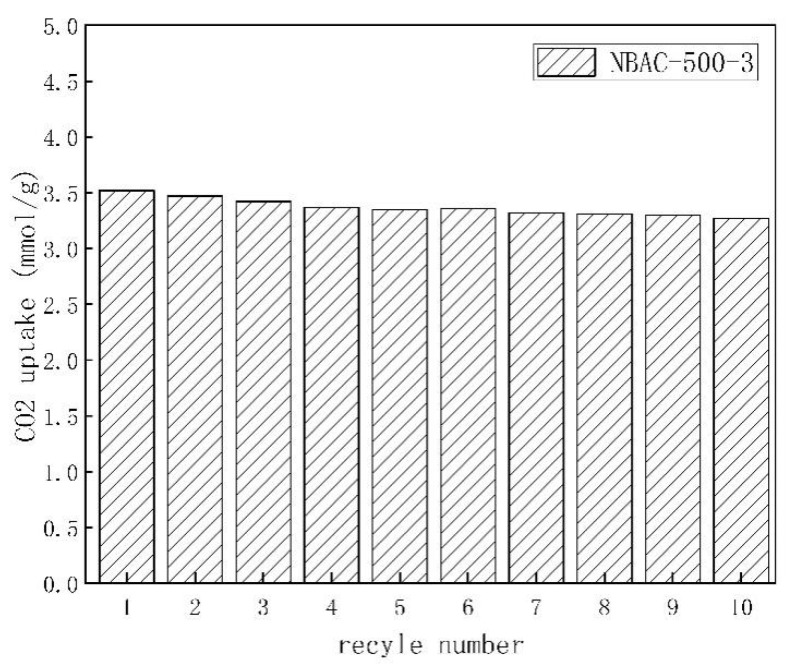
Adsorption/desorption experimental results for NBAC synthesized at 500 °C.

An analysis of reproducibility and steadiness of CO_2_ adsorption is essential for practical use of activated carbons. Five experimental cycles of adsorption/desorption were conducted to consider the usability at 1 bar and 25 °C. The results for those regenerated NBACs are shown in Figure 7. Approximately 93% of the adsorption capacity (3.27 mmol/g for the tenth cycle measurement as compared with 3.52 mmol/g for the virgin NBAC-500-3) was retained even after the 10-cycle measurement which aligned with the Qst results, suggesting that the dry chemically synthesized NBACs could be a perspective candidate for industrial use in CO_2_ adsorption and separation.

## 3. Experimental Section

### 3.1. Materials

Powder bamboo charcoal (40–60 mesh) was purchased from Zhejiang Wanlin Biotech Co, Ltd., Hangzhou, China, with pyrolysis at 750 °C for 7 days, the charcoal was oven-dried at 105 °C prior to use. Sodamide (SA, NaNH_2_) and hydrochloric acid (37%, HCl) were purchased from Shanghai Aladdin Biochemical Technology Co., Ltd., Shanghai, China. The reagents were used as received.

### 3.2. Synthesis of N-Doped Bamboo-Based Activated Carbon (NBAC)

The blended SA/BC samples (the blend ratio was set at 1:1, 2:1, and 3:1, respectively) were placed under an N_2_ atmosphere by applying a tube furnace (LTKC-8-16, Hangzhou Lantian Instrument Co., Ltd., Hangzhou, China), and the temperature was set at 400, 500, and 600 °C, for 2 h, respectively. Then, the raw N-doped bamboo-based activated carbon (NBAC) powders were obtained after cooling down to an ambient temperature. The NBAC samples were further rinsed using diluted hydrochloric acid (10%) to neutralize the residue and resultant of the activation and remove possible ash in the bamboo charcoal samples. The samples were termed as NBAC-x-y, where x refers to the activation temperature and y the blend ratio of NaNH_2_/BC.

### 3.3. Characterization

The surface morphologies of the samples were observed by field emission scanning electron microscopy (SEM, Hitachi SU 8010, Tokyo, Japan) at the emission voltage of 5 KV. The synthesized samples were sprayed with gold prior to observation. The elements (C, H and N) were measured by elemental analyzer (EA, Vario EL cube, Germany Elementary, Hesse, Germany) applying CHN mode. The specific surface area (SSA), as well as pore volume and pore size distribution were determined by an automated adsorption system (ASAP 2020, Micromeritics, Norcross, GA, USA) using the Brunauer–Emmett–Teller (BET) equations with nitrogen gas physisorption at 77 K. The surface elemental compositions were analyzed by X-ray photoelectron spectroscopy (XPS, Thermo Scientific K-Alpha, Waltham, MA, USA) with primary photon energies of 1486.6 eV.

### 3.4. CO_2_ Adsorption Measurement and Calculation

All NBAC samples were vacuum degassed at 300 °C for 6 h prior to adsorption measurement, followed by CO_2_ adsorption isotherm measurements at a pressure of 1.0 bar and temperatures of 0 °C and 25 °C. To evaluate the gas adsorption selectivity, the N_2_ adsorption isotherm of samples was also measured at 25 °C and pressure at 1 bar.

Adsorption heat and adsorption selectivity was calculated by the single site Langmuir–Freundlich equation (Equation (1)):

(1) $$q=\frac{q_{m}bp^{n}}{1+bp^{n}}$$

where *p* refers to the balancing pressure of gas expressed in MPa, *q* is the unit adsorption capacity of NBAC expressed in mmol, *q_m_* is the saturated adsorption capacity expressed in mmol, *b* is the affinity constant, and *n* is the index of heterogeneity.

Isosteric heat of adsorption was calculated using the Clausius–Clapeyron equation (Equation (2)):

(2) $$\ln\frac{P_{2}}{P_{1}}=-\frac{\Delta H}{R}\left(\frac{1}{T_{2}}-\frac{1}{T_{1}}\right)$$

where *P*_1_ and *P*_2_ refer to the relative pressure of the gas at *T*_1_ and *T*_2_, respectively, expressed in MPa; *T*_1_ and *T*_2_ refer to the temperature of 273 K (0 °C) and 295 K (25 °C), respectively; *R* is the ideal gas constant whose value is 8.314 J/(mol K); and Δ*H* is the enthalpy change of gas expressed in KJ/mol.

The adsorption selectivity of samples was calculated with the ideal adsorbed solution theory (IAST, Equation (3)):

(3) $$S=\frac{x_{1}/x_{2}}{y_{1}/y_{2}}$$

where *S* refers to the adsorption selectivity of binary gas mixture; *x*_1_ and *x*_2_ are the molar fractions of adsorbed CO_2_ and N_2_ in the NBAC sample, respectively; and *y*_1_ and *y*_2_ are the molar fractions of CO_2_ and N_2_ in the binary gas phase, respectively.

## 4. Conclusions

In summary, in this work, an in situ dry chemical synthesis was employed to fabricate N-doped bamboo-based activated carbon (NBAC) from conventional bamboo charcoal applying sodamide as an activation material and nitrogen source with nitrogen protection at a medium temperature (400–600 °C) in this work. The as-synthesized NBAC with high nitrogen content and narrowly distributed micropores presented a specific surface area with 756–1489 m^2^/g, excellent CO_2_ adsorption performance, Among all the synthesized samples, NBACs obtained at 500 °C with a sodamide/bamboo charcoal blend ratio of 3:1, demonstrated the highest CO_2_ adsorption of 4.95 mmol/g at 0 °C and 1 bar, fairly good CO_2_/N_2_ adsorption selectivity, low isosteric heat of adsorption, and good recycling and regeneration performance, which made the NBAC a candidate absorbent in CO_2_ capture and utilization.

## Figures and Tables

**Table 1:** Pore parameters of NBACs synthesized from N-doping activation.

Absorbent	S_BET_ (m^2^/g)	V_tot_ (cm^3^/g)	V_mic_ (cm^3^/g)	V_mic_/V_tot_ (%)	V_(0.33–1 nm)_ (cm^3^/g)
NBAC-400-1	756	0.300	0.271	90.33%	0.219
NBAC-400-2	927	0.370	0.322	87.03%	0.229
NBAC-400-3	1068	0.450	0.381	84.67%	0.241
NBAC-500-1	1025	0.420	0.369	87.86%	0.271
NBAC-500-2	1175	0.506	0.426	84.19%	0.285
NBAC-500-3	1286	0.631	0.508	80.51%	0.282
NBAC-600-1	1227	0.543	0.441	81.22%	0.276
NBAC-600-2	1458	0.675	0.500	74.07%	0.281
NBAC-600-3	1489	0.682	0.480	70.38%	0.233

**Table 2:** Elemental content of NBACs synthesized by N-doping activation.

Absorbent	N (wt%)	C (wt%)	H (wt%)
BC	0.26	83.5	3.12
NBAC-400-1	3.25	71.2	2.98
NBAC-400-2	3.89	72.6	3.05
NBAC-400-3	4.12	72.9	2.79
NBAC-500-1	2.51	73.2	2.43
NBAC-500-2	2.85	75.1	2.34
NBAC-500-3	3.21	74.6	2.05
NBAC-600-1	1.98	76.2	1.78
NBAC-600-2	2.15	77.3	1.81
NBAC-600-3	2.35	78.5	1.69

**Table 3:** CO_2_ and N_2_ adsorption capacity for NBACs at 1 bar, at 0 °C and 25 °C.

Absorbent	CO_2_ Uptake (mmol/g)		N_2_ Uptake (mmol/g)
	0 °C	25 °C	25 °C
NBAC-400-1	3.68	2.49	0.33
NBAC-400-2	3.78	2.68	0.35
NBAC-400-3	3.85	2.91	0.36
NBAC-500-1	4.41	3.16	0.45
NBAC-500-2	4.71	3.41	0.40
NBAC-500-3	4.95	3.52	0.41
NBAC-600-1	4.48	3.05	0.49
NBAC-600-2	4.31	3.21	0.45
NBAC-600-3	3.76	2.78	0.46
Commercial BAC#1	/	1.43	/
Commercial BAC#2	/	1.87	/
Commercial BAC#3	/	2.21	/

**Table 4:** Fitting results and adsorption selectivity for NBACs synthesized at 500 °C.

Absorbent	Adsorbate	*q_m_*	*b*	*n*	R^2^	Selectivity
NBAC-500-1	CO_2_	8.02	0.65	0.772	0.99	15.03
	N_2_	2.51	0.219	0.943	0.99	
NBAC-500-2	CO_2_	7.47	0.84	0.865	0.99	16.87
	N_2_	2.23	0.218	0.946	0.99	
NBAC-500-3	CO_2_	7.76	0.83	0.85	0.99	16.97
	N_2_	1.35	0.436	0.936	0.99	

## Data Availability

The data presented in this study are available on request from the corresponding authors.
